# Supplementary material for: Burden and determinants of self-reported high blood pressure among women of reproductive age in Tanzania: Evidence from 2022 Tanzania demographic and health survey
Source: PLoS One. 2025 Feb 28;20(2):e0314901. doi: 10.1371/journal.pone.0314901 (PMC11870381; doi:10.1371/journal.pone.0314901)
Supplement: Attachment — Submitted filename: reviewers comments.pdf [file pone.0314901.s002.pdf]

Dear Editors and Authors,

Thank you for giving me the opportunity to review the manuscript, titled “Burden and Determinants of Self-Reported High Blood Pressure Among Women of Reproductive Age In Tanzania: Evidence from 2022 Tanzania Demographic and Health Survey”.

The authors of the study chose a very important topic related to high blood pressure (HBP) in reproductive age women. The authors used data from over 15,000 women from the Tanzania DHS 2022, and by employing a binary logistic regression model, the authors assessed predictors of self-reported HBP. They found that 6.6% of women, with mean age of 29.3 years, had HBP. In terms of predictors of HBP, older age women had higher BP, women with secondary education had higher BP, married and widow women had higher BP, richer women had higher BP, and women with self-reported poor health and recent visits to health facilities had higher BP.

My assessment of the manuscript is that it is well written and is well organized. The findings from this study have the potential to impact health interventions and policy to improve clinical guidelines on BP management and control, and to improve women’s health as a whole. However, the authors need to address the following issues before the paper can be considered for publication.

Abstract

The word “bracket” can be changed to “group” here, and elsewhere in the manuscript.

In the conclusion, it says “This study highlights the substantial burden of …”, and I’m not sure whether the 6.6% burden is substantial. The authors may opt to delete the word “substantial”.

Introduction

The introduction section starts well; however, when it narrows down on identifying the research gap, it fails to provide relevant and convincing evidence to justify the study, which is to examine the prevalence and predictors of HBP in women of reproductive health who are mainly young women 15-49 years old. Considering HBP is more prevalent in older people, mainly 50+ years, the authors need to provide strong justification for choosing this study population and examining HBP among them.

Methods

Under “study population and sample size” the authors should provide some narrative on what the study power is, using the sample size of 15254 women.

If there is a question on use of anti-hypertensive medications in the TDHS2022, the authors may use it to define their outcome, in the addition to the question “whether a participant reported having HBP”.

Results

Under “burden and determinants of self-reported HBP” the authors do not need to report p-values for aORs. However, aORs (95%CI) are enough in the narrative, and aORs (95%CI) and p-values can be kept in the tables. In Table 2, the authors should report aORs for “Wealth status”, Smoke cigarette, Self-report health status, Birth in last 5-years, because currently the authors have deleted them.

Discussion

The discussion needs to start from “The study found a 6.6% prevalence of ….”. Please delete the sentences about this, as they are unnecessary and redundant.

Under “strengths and limitations”, please start by stating the strengths of the study first; then write the limitations. One of the limitations that the authors did not discuss is the lack of data on BMI (or other measures of obesity), on dietary habits, diabetes, and on daily exercise. These are important confounders of HBP. Furthermore, the authors stated that this sampled population may genuinely represent the Tanzania population. I do not think this is correct, given the sample covers only women 15-49 years old, with mean age 29.3 years. The sample seems to be from a younger population, and we know that HBP is more prevalent in older people (e.g., 50+ years). In addition, this sample covers only women. There is evidence that sex is associated with HBP. Thus, this sample cannot be a reprehensive sample of the Tanzania population. The authors should acknowledge this as a limitation.
