# Supplementary material for: Burden and determinants of self-reported high blood pressure among women of reproductive age in Tanzania: Evidence from 2022 Tanzania demographic and health survey
Source: PLoS One. 2025 Feb 28;20(2):e0314901. doi: 10.1371/journal.pone.0314901 (PMC11870381; doi:10.1371/journal.pone.0314901)
Supplement: Attachment — Submitted filename: comments on PONE-D-24-35959.docx [file pone.0314901.s003.docx]

Dear Authors,

The manuscript discussed the prevalence and determinants of self-reported high blood pressure among women of reproductive age in Tanzania. The authors provided good information about the prevalence and the determinants of HBP based on the data from a national population-based survey with good number of samples. However, the authors need to revise the manuscript as below points to improve the quality of this paper.

Introduction

1- Please add a paragraph on why it is important to investigate the prevalence of high blood pressure among women of reproductive age. Why you chose this age group? And please provide a literature review of similar studies about prevalence and determinants of high blood pressure about women of reproductive age.

Result

1- In table 1, you can delete the frequency and percent for answer “No” to the self-reported high BP. Presenting data for yes, can show the frequency and percentage for “no” answer as well. I have the same comment for table 2. I recommend you present the bivariate analysis results in table 1 and the results of multivariate regression analysis in table 2.

Discussion

1- I recommend comparing the result of your study with the result of studies that reported the prevalence of high blood pressure in the same age group of female patients in Tanzania. It will show the difference between prevalence based on self-reported HBP and health facility reported HBP. In addition, I recommend discussing more on the trend of HBP prevalence among women till 2030 and connect your recommendations to the Sustainable Development Goals.
